# Intravitreal anti-vascular endothelial growth factor injections in pregnancy and breastfeeding: a case series and systematic review of the literature

**DOI:** 10.1038/s41433-023-02811-6

**Published:** 2023-11-18

**Authors:** Ariel Yuhan Ong, Christine A. Kiire, Charlotte Frise, Yasmin Bakr, Samantha R. de Silva

**Affiliations:** 1 Oxford Eye Hospital, Oxford University Hospitals NHS Foundation Trust, Oxford, UK; 2 Stoke Mandeville Hospital, Buckinghamshire Healthcare NHS Trust, Aylesbury, UK; 3 Queen Charlotte’s and Chelsea Hospital, Imperial College Healthcare NHS Trust, London, UK; 4 Nuffield Department of Clinical Neurosciences, University of Oxford, Oxford, UK

**Keywords:** Retinal diseases, Adverse effects

## Abstract

**Introduction:**

Anti-vascular endothelial growth factor (anti-VEGF) agents may occasionally need to be considered for sight-threatening macular pathology in pregnant and breastfeeding women. This is controversial due to the dearth of data on systemic side effects for mother and child. We aimed to expand the evidence base to inform management.

**Methods:**

Retrospective case series of pregnant and breastfeeding women treated with intravitreal anti-VEGF injections at Oxford Eye Hospital between January 2015 and December 2022. In addition, we conducted a systematic review and combined eligible cases in a narrative synthesis.

**Results:**

We treated six pregnant women with anti-VEGF for diabetic macular oedema(DMO) (*n* = 5) or choroidal neovascularisation (CNV) (*n* = 1). Four received ranibizumab whilst two (not known to be pregnant) received aflibercept. Patients known to be pregnant underwent counselling by an obstetric physician. Five pregnancies resulted in live births. Combining our cases with those previously published, treatment of 41 pregnant women (42 pregnancies) are reported. Indications for treatment included CNV (*n* = 28/41,68%), DMO (*n* = 7/41,17%) and proliferative diabetic retinopathy (*n* = 6/41,15%). Bevacizumab (*n* = 22/41,54%) and ranibizumab (*n* = 17/41,41%) were given more frequently than aflibercept (*n* = 2/41,5%). Many (*n* = 16/41,40%) were unaware of their pregnancy when treated. Most pregnancies resulted in live births (*n* = 34/42,81%). First trimester miscarriages (*n* = 5/42,12%) and stillbirths (*n* = 3/42,7%) mostly occurred in women with significant risk factors.

**Conclusion:**

Intravitreal anti-VEGF injections may not necessarily compromise obstetric outcomes, although clear associations cannot be drawn due to small numbers and confounders from high rates of first trimester miscarriages in general and inherently high-risk pregnancies. It may be worth considering routinely investigating pregnancy and breastfeeding status in women of childbearing age prior to each injection, as part of anti-VEGF treatment protocols.

## Introduction

The management of sight-threatening macular pathology in pregnant women is challenging. Conditions such as diabetic macular oedema (DMO) may progress with physiological changes in pregnancy [1], and while this may regress in the postpartum period, other conditions such as choroidal neovascularization (CNV) do not, and may result in permanent structural damage and sight loss if left untreated. Therefore, treatment with intravitreal anti-vascular endothelial growth factor (anti-VEGF) agents must occasionally be considered. While anti-VEGF injections are effective, their use in pregnant women is controversial due to the paucity of data on systemic side effects for mother and child [2, 3]. This poses a conundrum for ophthalmologists, particularly given that most will have limited experience due to the relative infrequency of this situation, and the lack of guidelines on managing and counselling pregnant patients [1]. Patient anxiety over competing risks to their vision versus their child may further compound this difficult situation.

There are even less data on the extent to which intravitreal anti-VEGF agents transfer to breast milk, and the potential consequences for breastfed infants. Women who defer anti-VEGF treatment until after delivery may therefore prefer to forego breastfeeding due to concerns about potential effects on their child [4]. Breastfeeding carries both short-term and long-term benefits in terms of nutrition, immunity, cognitive development, and limiting the risk of developing chronic systemic diseases [5, 6]. Avoiding unnecessary cessation would be in the best interests for women who wish to breastfeed and their babies.

While animal studies have demonstrated adverse effects of anti-VEGF drugs in pregnancy, robust research has not been adequately conducted in humans. Without adequate experience and evidence, macular pathology in pregnant women may be undertreated, and the potential benefits to the mother overshadowed by a hesitancy to treat based on theoretical risk to the fetus.

We report outcomes and adverse events from a case series and systematic review of anti-VEGF injections in pregnant and breastfeeding women, with the aims of expanding the evidence base, informing real world clinical practice, and highlighting key issues for future research.

## Methods

### Case series

We undertook a retrospective review of consecutive pregnant patients treated with intravitreal anti-VEGF injections at the Oxford Eye Hospital between January 2015 and December 2022. Patients were identified via the departmental electronic medical record system (Medisoft, Leeds, United Kingdom), and were included if they had received at least one intravitreal anti-VEGF injection during pregnancy and/or whilst known to be breastfeeding. This work was registered as a clinical audit at the Oxford University Hospitals NHS Foundation Trust (audit number: 8268) and did not require formal ethical approval.

Clinical records were reviewed for demographic information; ocular and systemic comorbidities (including risk factors for miscarriage); referral pathway to the ophthalmology department; clinical presentation (indication for treatment, laterality, visual acuity); anti-VEGF treatment (drug and dose given, number of injections given and gestational age at each injection); complications (pregnancy outcome, and ocular, obstetric, and neonatal complications). Pregnancy status (i.e. whether the patient was known to be pregnant) at the time of injection was recorded. Documentation of whether counselling took place prior to commencing anti-VEGF injections and who performed this counselling was also reviewed.

### Systematic review

A PubMed search was subsequently conducted on 8 April 2023. Key search terms were divided into two categories, combined with a Boolean operator: (anti-VEGF OR bevacizumab OR ranibizumab OR aflibercept OR pegaptanib OR conbercept OR brolucizumab OR faricimab OR intravitreal injection) AND (pregnan* OR breastfe* OR lactat* OR breast milk OR postpartum). Animal studies were excluded. No date limits were applied. Reference lists of reviews and included studies were examined for further potentially relevant studies.

All study types above the level of expert commentary (level 4 evidence and above, as defined by the Oxford Centre for Evidence-based Medicine [7]) were eligible for inclusion. Cases of women receiving intravitreal anti-VEGF treatment during pregnancy or while breastfeeding were included if they reported the following outcomes to allow synthesis of results: indication for treatment, anti-VEGF agent and number of injections administered, gestational age at treatment, pregnancy outcome, risk factors for miscarriage, and obstetric and neonatal complications.

Abstracts and full texts were screened for eligibility by two independent authors using Rayyan web software. Any discrepancies were resolved through discussion or arbitrated by a third author if consensus could not be reached.

Eligible cases identified from the systematic review were combined with our case series. Continuous data were described by mean and standard deviation (SD). No statistical analyses were planned because of the small numbers precluding meaningful interpretation.

## Results

### Case Series

#### Intravitreal anti-VEGF injections in Pregnancy

We included six women treated with intravitreal anti-VEGF injections during pregnancy. Patient characteristics are described in Table 1. Indications for treatment included centre-involving DMO (*n* = 5) (with central subfield thickness of >400 µm on optical coherence tomography (OCT) imaging and disabling symptoms) and myopic CNV (*n* = 1).

**Table 1:** Clinical summary of patients who received intravitreal anti-VEGF treatment in pregnancy in our case series.

ID	Indication	Age (years)	Intravitreal Anti-VEGF given	Eye	No.	Gestation (weeks) at injection	Risk factors for miscarriage	Obstetric Complications	Pregnancy Outcome	Neonatal/ Child Complications	Counselling	Baseline VA	VA 4 weeks after IVI	Last known VA	Follow-up (month)	Ocular Comorbidities
1	Myopic CNV	39	Ranibizumab 0.25 mg	Right	3	26, 30, 36	Maternal age ≥ 35, BMI > 25	None	Live birth	Unknown	Medical retina consultant	0.74	0.40	0.70	77	Myopic macular degeneration, Scleral buckle for RRD
2	DMO	31	Aflibercept 2 mg	Right	3	2, 15, 21	T1DM, ESRD (Dialysis)	IUGR (1005 g)	Live birth	Preterm delivery (29 ^+ 6^ weeks), reached developmental milestones as of 4 years	Not known to be pregnant at time of injection; later counselled by vitreoretinal team	0.50	0.60	0.48	58	PPV/Delam/SO for TRD
3	DMO	37	Ranibizumab 0.5 mg	Both	2	21 (Bilat)	T1DM, ESRD (Dialysis), Maternal age ≥ 35,	PPROM	Stillbirth 3 weeks after IVI (PPROM)	N/A	Medical retina + Obstetric medicine consultants	0.30 R, 0.30 L	0.40 R, 0.30 L	0.40 R, 0.30 L	39	NPDR
4	DMO	27	Ranibizumab 0.5 mg	Right	2	20, 24	T1DM, CKD	Pre-eclampsia, IUGR (1005 g)	Live birth	Preterm delivery (29^ + 3^ weeks), initial failure to thrive but no further concerns at 22 months	Obstetric medicine consultant	1.08	1.18	2.70	24	Concurrent high-risk PDR treated with PRP during pregnancy
5	DMO	32	Ranibizumab 0.5 mg	Both	2	34 (Right), 36 (Left)	T1DM	Pre-eclampsia/ HELLP syndrome	Live birth	No concerns at 22 months	Obstetric Medicine consultant	0.40 R, 0.40 L	0.30 R, 0.40 L	0.20 R, 0.00 L	22	PDR
6	DMO	37	Aflibercept 2 mg	Both	2	13 (Bilat)	T2DM, Maternal age ≥ 35	Pre-eclampsia, IUGR (1356 g)	Live birth	Preterm delivery (32 ^+ 3^ weeks), reached developmental milestones as of 10 months	Not known to be pregnant at time of injection; no further injections given	0.48 R, 0.78 L	NR	0.30 R, 0.30 L	15	NTG, RE trabeculectomy & complicated cataract surgery

*CNV* choroidal neovascularisation, *DMO* diabetic macular oedema, *VEGF* anti-vascular endothelial growth factor, *BMI* body mass index, *ESRD* end stage renal disease, *CKD* chronic kidney disease, *IUGR* intrauterine growth restriction, *PPROM* preterm premature rupture of membranes, *HELLP* haemolysis elevated liver enzymes low platelets, *IVI* intravitreal injection, *VA* visual acuity, *RRD* rhegmatogenous retinal detachment, *TRD* tractional retinal detachment, *NPDR* non-proliferative diabetic retinopathy, *PDR* proliferative diabetic retinopathy, *NTG* normal tension glaucoma.

##### Referral route

Three patients were already under the retinal clinic for pre-existing conditions. One patient was referred by the obstetrics team following admission for pregnancy-related complications (proteinuria, renal dysfunction, and severe fluid overload on a background of poorly controlled diabetes); another from the diabetic screening service; and a further patient from the independent sector for further treatment for DMO and consideration of cataract surgery.

##### Anti-VEGF treatment

Nine eyes of 6 patients were treated with 2.3 (SD 0.5) injections during pregnancy (Table 1). Treatment initiation was evenly distributed among the first, second, and third trimesters (two patients each). The two patients treated in the first trimester were not known to be pregnant at the time, and were given aflibercept. Four patients known to be pregnant received ranibizumab, which was chosen due to its lower systemic absorption. Of these, one received half-dose treatment (0.25 mg) due to concerns about potential fetotoxicity and had a partial response to treatment. One patient with DMO received periocular triamcinolone with a partial treatment response prior to being treated with anti-VEGF.

**Fig. 1:**
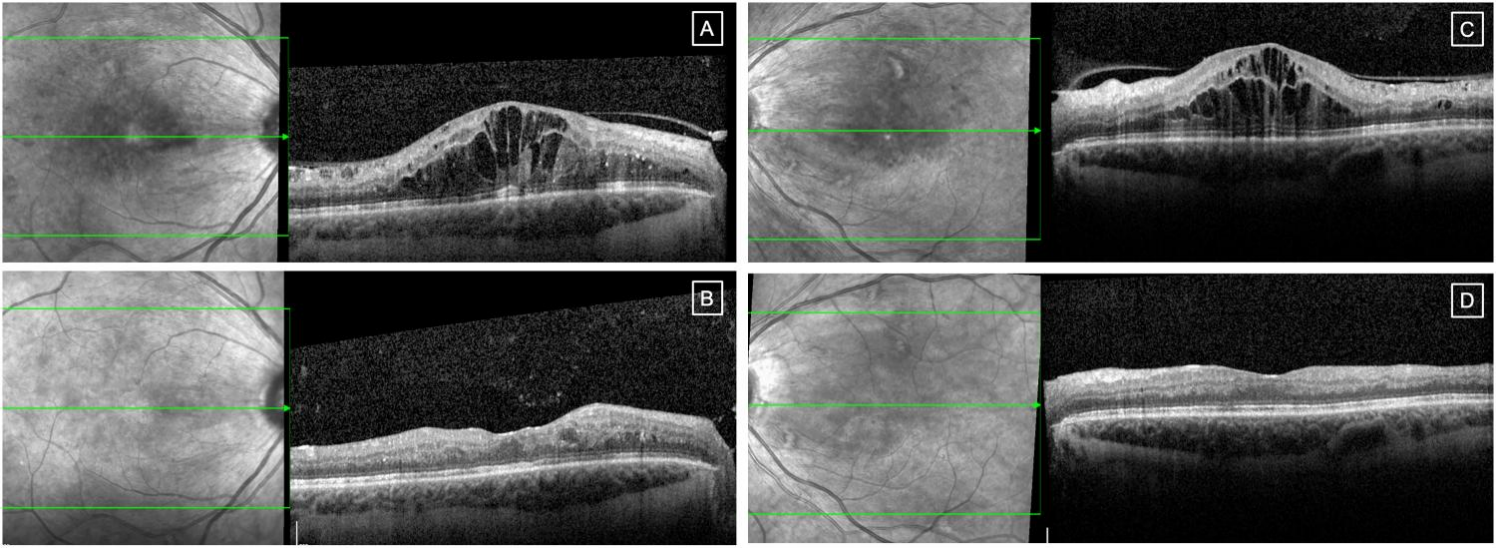
Imaging of the macula in patient #5, demonstrating good treatment response to anti-VEGF injections in diabetic macular oedema. Near-infrared reflectance (left hand image) and OCT (right hand image) in the right eye pre-treatment (**A**) and 4 weeks post-treatment (**B**); and in the left eye pre-treatment (**C**) and 4 weeks post-treatment (**D**).

Mean visual acuity (VA) was 0.53 (SD 0.28) LogMAR at baseline, and 0.51 (SD 0.31) approximately 4 weeks after the prescribed course of treatment. No intraocular complications such as inflammation or endophthalmitis were observed. Patients demonstrated good treatment response and subjective improvement in symptoms 4 weeks after receiving anti-VEGF injections (example: Fig. 1), although objective VA gains were limited by structural changes on OCT such as retinal thinning and disorganisation of the inner retinal layers that became apparent after the severe DMO had resolved. Several patients had multiple ocular comorbidities affecting final visual potential, as detailed in Table 1.

##### Pre-treatment counselling

For the four patients known to be pregnant, multidisciplinary discussions regarding material risks and benefits of all potential treatment options were conducted between their consultant ophthalmologist and consultant obstetric physician and/or maternal/fetal medicine obstetrician after they were diagnosed with macular pathology eligible for anti-VEGF treatment. Patients were subsequently counselled about the risks associated with the pregnancy, particularly in the context of systemic comorbidities such as pre-existing diabetes, as well as the potential impact of anti-VEGF injections on these risks. Once fully informed about treatment options and alternatives (e.g. observation only, a trial of periocular corticosteroid injections or intravitreal corticosteroids, as appropriate), the patients consented to treatment with anti-VEGF injections.

##### Pregnancy outcomes and obstetric complications

Five of six pregnancies resulted in live births, of which four were complicated by pre-eclampsia, premature delivery, and/or intrauterine growth restriction (IUGR). All complications occurred in patients with pre-existing diabetes, with risk factors for poor pregnancy outcomes such as poor glycaemic control, high body mass index, and older maternal age. One patient experienced a stillbirth at 24 weeks (from preterm premature rupture of membranes with subsequent sepsis), three weeks after receiving bilateral ranibizumab injections for DMO. This patient had multiple risk factors for a poor obstetric outcome including cervical insufficiency, end-stage renal disease requiring dialysis, older maternal age, and poor glycaemic control. There had been serious concerns about the prognosis of her pregnancy even before anti-VEGF treatment.

##### Neonatal and developmental complications

Three premature neonates required further care in the neonatal intensive care unit (NICU), but no further adverse neonatal events were observed. These children were born to mothers with poorly controlled diabetes, and there was no observable pattern with a specific anti-VEGF agent or the trimester at which treatment was administered. No developmental issues were identified in the children who were between 8 and 74 months of age at the time of writing.

#### Intravitreal anti-VEGF injections in breastfeeding women

One patient had a single intravitreal bevacizumab injection at 8 weeks postpartum while continuing to breastfeed. This was administered intraoperatively during pars plana vitrectomy and delamination for tractional retinal detachment secondary to proliferative diabetic retinopathy (PDR). She had been offered additional panretinal photocoagulation for high-risk PDR and a range of treatment options for severe DMO during her pregnancy, and despite counselling, especially with regards to progression of PDR in pregnancy, had declined any intervention until after delivery due to concerns about potential treatment risks. No ocular or neonatal complications were observed up to 12 months post-injection.

### Systematic review results

**Fig. 2:**
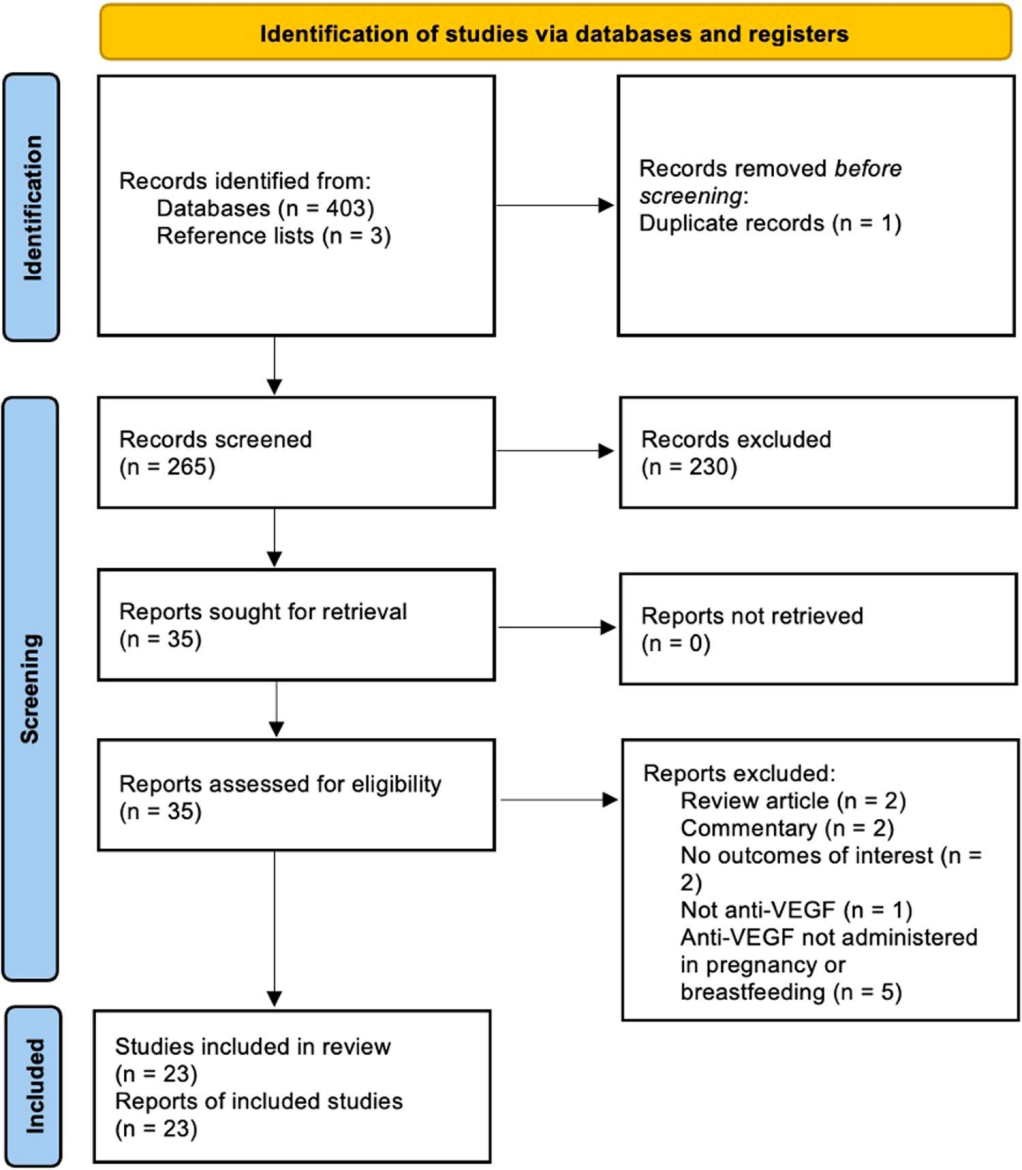
PRISMA flow diagram for the systematic review.

The systematic search produced 403 potentially relevant records. Following abstract screening, 35 full texts were reviewed, resulting in 23 articles eligible for inclusion (Fig. 2).

#### Intravitreal anti-VEGF injections in Pregnancy

With the addition of our case series, this systematic review comprises 41 women (42 pregnancies) treated with anti-VEGF injections during pregnancy (Table 2). The most common indication for treatment was CNV (*n* = 28, 68%) from myopia (*n* = 7), punctate inner choroidopathy (*n* = 7), presumed ocular histoplasmosis syndrome (*n* = 6), idiopathic (*n* = 4), multifocal choroiditis (*n* = 1), and sarcoid uveitis (*n* = 1). This was followed by DMO (*n* = 7, 17%) and PDR (*n* = 6, 15%).

**Table 2:** Summary of cases receiving intravitreal anti-VEGF treatment in pregnancy in the literature.

Author, Date	Indication	Intravitreal Anti-VEGF	No.	Gestation at injection	Age (years)	Risk factors for adverse pregnancy outcomes	Obstetric Complications	Pregnancy Outcome	Neonatal/ Child Complications	Known to be pregnant
2023	Myopic CNV	Ranibizumab 0.25 mg	3	26, 30, 36 weeks	39	Maternal age ≥ 35, BMI > 25	None	Live birth	NR	Yes
2023	DMO	Aflibercept 2 mg	3	2, 15, 21 weeks	31	T1DM, ESRD (Dialysis)	IUGR (1005 g)	Live birth	Preterm delivery (29 ^+ 6^weeks), reaching developmental milestones	No
2023	DMO	Ranibizumab 0.5 mg	2	21 weeks (Bilateral)	37	T1DM, ESRD (Dialysis), Maternal age ≥ 35	PPROM	Stillbirth at 24 weeks (3 weeks after IVI) (PPROM)	N/A	Yes
2023	DMO	Ranibizumab 0.5 mg	2	20, 24 weeks	27	T1DM, CKD	Pre-eclampsia, IUGR (1005 g)	Live birth	Preterm delivery (29 ^+ 3^weeks), initial failure to thrive but no further concerns	Yes
2023	DMO	Ranibizumab 0.5 mg	2	34 weeks (Right), 36 (Left)	32	T1DM	Pre-eclampsia/ HELLP syndrome	Live birth	No concerns at 22 months	Yes
2023	DMO	Aflibercept 2 mg	2	13 weeks (Bilateral)	37	T2DM, Maternal age ≥ 35	Pre-eclampsia, IUGR (1356 g)	Live birth	Preterm delivery (32 ^+ 3^ weeks), reaching developmental milestones at 10 months	No
Akkaya, [30]	DMO	Ranibizumab 0.5 mg	1	5 weeks	24	T1DM	Miscarriage	Miscarriage at 6 weeks (6 days after IVI)	N/A	No
Capuano, [39]	Advanced PDR	Ranibizumab 0.5 mg	1	30 weeks	33	T1DM (poor control)	None	Live birth	NR	Yes
De Groot, [40]	CNV from MFC	Ranibizumab 0.5 mg	5	5, 11, 20, 25, 30 weeks	NR	Multiple previous miscarriages and intrauterine fetal death	Placental abruption	Stillbirth at 33 weeks (Placental abruption)	N/A	Unclear
De Groot, [40]	CNV from MFC	Ranibizumab 0.5 mg	2	30, 37 weeks	NR	NR	None	Live birth	No neonatal complications	Yes
De Groot, [40]	CNV from MFC	Ranibizumab 0.25 mg (32 weeks), 0.5 mg (36 weeks)	2	32, 36 weeks	NR	NR	None	Live birth	No neonatal complications	Yes
De Groot, [40]	CNV from PIC	Ranibizumab 0.5 mg	2	33, 37 weeks	NR	NR	None	Live birth	No neonatal complications	Yes
De Groot, [40]	CNV from PIC	Ranibizumab 0.5 mg	1	27 weeks	NR	NR	None	Live birth	No neonatal complications	Yes
De Groot, [40]	CNV from PIC	Bevacizumab 1.25 mg (2 weeks), Ranibizumab 0.5 mg (20 weeks)	2	2, 20 weeks	NR	NR	None. NB: Elective C-section (risk of subretinal haemorrhage from vaginal delivery)	Live birth	No neonatal complications	Unclear
Fossum, [41]	Idiopathic CNV	Ranibizumab 0.5 mg	2	10, 21 weeks	26	Previous miscarriage	None	Live birth	No neonatal complications	Unclear
,Fossum [41]	Myopic CNV	Ranibizumab 0.5 mg	1	17 weeks	31	None	None	Live birth	No neonatal complications	Unclear
Fossum, [41]	CNV from PIC	Ranibizumab 0.5 mg	1	8 weeks	30	‘Complex obstetric history’ not otherwise specified	Cholestasis of pregnancy at 36 weeks prompting induction at 38 weeks	Live birth	No neonatal complications	Unclear
,Gomez Ledesma [29]	CNV from POHS	Bevacizumab 1.25 mg	1	Within a few days of conception	41	Maternal age ≥ 35	Miscarriage	Miscarriage at 8 weeks (8 weeks after IVI)	N/A	No
Introini, [42]	Myopic CNV	Bevacizumab 1.25 mg	1	7 weeks	35	Maternal age ≥ 35	None	Live birth	No neonatal complications, reached developmental milestones up to 12 months	Yes, MDT counselling
Jouve, [43]	Idiopathic CNV	Ranibizumab 0.5 mg	1	8 months	29	NR	None	Live birth	No neonatal complications	Yes
Kianersi, [31]	DMO	Bevacizumab 1.25 mg	1	10 weeks	29	T1DM	Miscarriage	Vaginal bleeding 18 h after IVI, subsequent miscarriage ?when	N/A	No
Kianersi, [32]	Advanced PDR	Bevacizumab 1.25 mg	1	12 weeks	27	T1DM (poor control), Previous miscarriages, High BMI	Gestational hypertension, Intrauterine fetal death	Stillbirth at 24 weeks	N/A	No
Pencak, [44]	Myopic CNV	Ranibizumab 0.5 mg	1	36 weeks	34	None	None	Live birth	No neonatal complications	Yes, MDT counselling
Petrou, [8]	PDR with vitreous hge	Bevacizumab 1.25 mg	1	4 weeks	29	T1DM	Miscarriage	Miscarriage at 5 weeks (1 week after IVI)	N/A	No
Petrou, [8]	Myopic CNV	Bevacizumab 1.25 mg	1	3 weeks	25	None	Miscarriage	Miscarriage at 4 weeks (10 days after IVI)	N/A	No
Polizzi, [33]	DMO, PDR	Bevacizumab 1.25 mg	2	Within 5 ± 3 days of ovulation; 4^+2^ weeks	37	Maternal age ≥ 35, T1DM, Hypertension, Previous miscarriage	Elective C-section for suspected macrosomia (not present)	Live birth	Reached developmental milestones up to 24 months	No
Polizzi, [45]	Myopic CNV	Bevacizumab 1.25 mg	1	13^+6^ weeks	NR	Maternal age ≥ 35, previous miscarriage	None	Live birth	Reached developmental milestones	Yes
Polizzi, [45]	CNV from POHS	Bevacizumab 1.25 mg	2	2^nd^ & 3^rd^ trimesters	NR	None	None	Live birth	Reached developmental milestones	Yes
Polizzi, [45]	CNV from POHS	Bevacizumab 1.25 mg	2	3^rd^ trimester	NR	None	None	Live birth	Reached developmental milestones	Yes
Rosen, [46]	CNV from PIC	Bevacizumab 1.25 mg	1	3 months^a^	24	None	None	Live birth	No neonatal complications up to 3 months	Yes
Sarhianaki, [47]	Idiopathic CNV	Ranibizumab 0.5 mg	1	Start of 3^rd^ trimester	29	None	None	Live birth	No neonatal complications	Yes
Sarmad, [48]	Advanced PDR	Bevacizumab 1.25 mg	1	1 week	31	T1DM	None	Live birth	No neonatal complications	No^b^
Sullivan, [34]	Idiopathic CNV	Bevacizumab 1.25 mg	1	19 days	20	None	None	Live birth	No complications up to 18 weeks	No
Sullivan, [34]	CNV from PIC	Bevacizumab 1.25 mg	1	21 days	27	None	None	Live birth	No complications up to 6 weeks	No^c^
Sullivan, [34]	PDR	Bevacizumab 1.25 mg	1	24 days	20	DM	None	Live birth	No complications up to 11 months	No
Sullivan, [34]	PDR with NVG	Bevacizumab 1.25 mg	1	20 days	25	DM, Hypertension	C-section for pre-eclampsia, IUGR (1260 g)	Live birth	Pre-term delivery (29 weeks); intubated for respiratory distress and pulmonary hge; ICH; blood transfusion for abnormal clotting and anaemia of prematurity	No
Tarantola, [9]	CNV from sarcoid uveitis	Bevacizumab 1.25 mg	4	17, 21, 26, 31 weeks	31	NR	None	Live birth	No neonatal complications, reached developmental milestones up to 8 months	Yes
Tarantola, [9]	CNV from POHS	Bevacizumab 1.25 mg	6	1, 9, 14, 20, 26, 32 weeks	36	Maternal age ≥ 35	Macrosomia	Live birth	No neonatal complications, normal growth and development	No
Tarantola, [9]	CNV from PIC	Bevacizumab 1.25 mg	1	3 weeks	33	NR	None	Live birth	No neonatal complications, normal growth and development up to 12 months	Unknown
Tarantola, [9]^d^	CNV from POHS	Bevacizumab 1.25 mg	1	23 weeks	27	NR	None	Live birth	No neonatal complications, normal growth and development up to 23 months	Yes
	CNV from POHS	Bevacizumab 1.25 mg	1	36 weeks	28	NR	None	Live birth	No neonatal complications, normal growth and development up to 9 months	Yes
Wu, [35]	Myopic CNV	Bevacizumab 1.25 mg	1	2 weeks	25	None	None	Live birth	No complications up to 12 months	No

^a^patient received Verteporfin with PDT at 1–2 weeks gestation, prior to becoming aware of pregnancy.

^b^patient had a negative pregnancy test prior to IVI, but was subsequently found to be 5 weeks pregnant 4 weeks after the IVI.

^c^patient reported negative pregnancy test at the time of IVI, but was subsequently found to be pregnant.

^d^patient was treated during 2 separate pregnancies for a new onset CNV during the first pregnancy and a recurrence during the second.

*CNV* choroidal neovascularisation, *DMO* diabetic macular oedema, *VEGF* anti-vascular endothelial growth factor, *BMI* body mass index, *ESRD* end stage renal disease, *CKD* chronic kidney disease, *IUGR* intrauterine growth restriction, *PPROM* preterm premature rupture of membranes, *HELLP* haemolysis elevated liver enzymes low platelets, *IVI* intravitreal injection, *MFC* multifocal choroiditis, *PIC* punctate inner choroidopathy, *POHS* presumed ocular histoplasmosis syndrome, *DM* diabetes mellitus.

These patients received a mean of 1.7 (SD 1.2) intravitreal anti-VEGF injections during their pregnancies. Three patients (from our case series) underwent bilateral injections for DMO; the remainder were unilateral. Bevacizumab (*n* = 22, 54%) and ranibizumab (*n* = 17, 41%) were given more frequently than aflibercept (*n* = 2, 5%), and one patient received both bevacizumab and ranibizumab during her pregnancy.

Treatment was most often initiated in the first trimester (*n* = 24, 57%) followed by the third (*n* = 12, 29%) and second trimesters (*n* = 6, 14%). Forty percent (*n* = 16) of patients were unaware of their pregnancy at the time of treatment, and were therefore inadvertently treated without appropriate counselling in the first trimester at a mean of 4 (SD 4) weeks, with two patients receiving treatment within a few days of the presumed conception date. Most (*n* = 14/16, 88%) did not have further anti-VEGF injections following the discovery of their pregnancy.

Most (*n* = 34, 81%) pregnancies resulted in live births, of which five were complicated by pre-eclampsia, premature delivery, and/or IUGR, all in women with risk factors for adverse outcomes such as pre-existing diabetes and/or poor glycaemic control. The remainder were first trimester miscarriages (n = 5, 12%) or stillbirths in women with complex obstetric histories (*n* = 3, 7%). In women who had no risk factors for adverse pregnancy outcomes, there was only one case of very early pregnancy loss at 4 weeks’ gestation [8].

No adverse neonatal events were reported beyond the NICU admissions for preterm delivery and sequelae of IUGR.

#### Intravitreal anti-VEGF injections in breastfeeding women

Six women received anti-VEGF injections while continuing to breastfeed (Table 3), including one from our case series. Treatments used were ranibizumab (*n* = 3), bevacizumab (*n* = 2), and conbercept (*n* = 1). One patient started treatment with bevacizumab injections during breastfeeding which continued into a subsequent pregnancy (included above) [9]. No data on adverse events in breastfed children were available apart from the one in our case series who remained well 12 months later.

**Table 3:** Summary of breastfeeding women receiving intravitreal anti-VEGF treatment in the literature.

Author, Date	Indication	Intravitreal Anti-VEGF	#	Timing of anti-VEGF injection(s)	Maternal Age (years)	Neonatal/ Child Complications
2023	Advanced PDR	Bevacizumab 1.25 mg	1	8 weeks postpartum	30	No complications up to 12 months
Groselli, [49]	CMO from CRVO	Ranibizumab 0.5 mg	>4	Starting 3 weeks postpartum, treat and extend regimen over 12 months	40	NR
Huang, [22]^a^	Idiopathic CNV	Ranibizumab 0.5 mg	4	Starting 6 months postpartum	30	NR
Juncal, [50]	Myopic CNV	Ranibizumab 0.5 mg	2	0 and 4 weeks postpartum	37	NR
Shao, [4]	Idiopathic CNV	Conbercept 0.05 mg	4	2, 3, 4, 7 months	27	NR
Tarantola, [9]^b^	CNV from PIC	Bevacizumab 1.25 mg	5	Unknown	33	NR

^a^Patient stopped breastfeeding for 3 days after each injection (while continuing to express and discard milk).

^b^Patient became pregnant again and received 1 further anti-VEGF injection in her next pregnancy (see Table 2).

*PDR* proliferative diabetic retinopathy, *CMO* cystoid macular oedema, *CRVO* central retinal vein occlusion, *CNV* choroidal neovascularisation, *NR*not recorded, *VEGF* vascular endothelial growth factor.

## Discussion

We report outcomes from a case series and systematic review of 41 women (42 pregnancies) who were treated with intravitreal anti-VEGF injections, and 6 women who received anti-VEGF injections while breastfeeding.

### Safety concerns: obstetric complications

It was not possible to draw conclusions about associations between anti-VEGF treatment and obstetric complications due to the low numbers of reported cases and confounders from early-term miscarriages and inherently high-risk pregnancies in women with pre-existing diabetes. Data from our systematic review (Table 2) suggests that of the 20 patients without any known risk factors for adverse pregnancy outcomes, only one experienced a miscarriage (at 4 weeks gestation). At the same time, rates of early pregnancy losses can approach 31% in healthy women before pregnancy is recognised [10]. High quality real-world data from prospective multicentre studies would be helpful for exploring this further, to investigate the safety signal and potentially reduce the risk of undertreating pregnant women who might benefit from this treatment.

There are insufficient data on which anti-VEGF agent is safest in pregnancy. Ranibizumab and bevacizumab were given more frequently than aflibercept in our study. Ranibizumab has the lowest systemic absorption and shortest half-life (5.8 (SD 1.8) days). Bevacizumab has a relatively high systemic exposure and longer half-life. Aflibercept causes the greatest reduction in serum free VEGF relative to baseline levels [11]. It is unclear whether the higher systemic drug exposure after intravitreal dosing would be significant for developing fetuses, or whether this may be more relevant for intravenous treatment. In addition, there are insufficient safety data to recommend an optimal time point at which anti-VEGF injections can be safely administered in pregnancy. Where possible, avoiding anti-VEGF treatment during the first trimester may be advisable, due to the theoretically higher risk of teratogenic effects in early pregnancy. The two patients from our case series who were treated in their first trimester (both having received aflibercept) were not known to be pregnant at the time. These two pregnancies were complicated by premature delivery and IUGR in the context of poor glycaemic control, but without subsequent reported neonatal adverse effects.

Two database studies were excluded from our analysis because few of our specified outcomes were reported, and only study-level data were available which were not amenable to data synthesis [12, 13]. Sakai et al. was a pharmacovigilance study which described adverse events in pregnant women treated with intravitreal anti-VEGF injections identified from the United States FDA Adverse Events Reporting System (FAERS) database [12]. Pregnancy loss was reported in 19 cases treated with ranibizumab, 6 cases with bevacizumab, and 4 cases with aflibercept. However, FAERS does not contain data on intravitreal anti-VEGF injections in those with uncomplicated pregnancies, risk factors for poor obstetric outcomes, stage of pregnancy at which treatment was initiated, or when pregnancy loss occurred. Limitations of this approach also include the possibility of duplicate or incomplete reports, lack of verification, and potential positive reporting bias [14].

Ben Ghezala et al. reported results from a retrospective cohort study in France between 2009–2018, comparing obstetric and neonatal complications in pregnant women admitted to hospital who had received intravitreal anti-VEGF versus corticosteroid injections [13]. One hundred pregnant women received anti-VEGF injections during their pregnancy or in the preceding month, with ten pregnancy losses and 23 terminations of pregnancy among this cohort. No data were available to explain whether the terminations were undertaken because of the risk of maternal comorbidities being exacerbated by pregnancy or because of potential fetotoxicity from anti-VEGF injections. The anti-VEGF agent given was not specified, and individual level data on risk factors for obstetric complications and stage of pregnancy were not available. In addition, miscarriages managed on an outpatient basis were not captured. Obstetric and neonatal complications (including abnormal fetal heart rate, neonatal distress, and prematurity) were comparable between corticosteroid and anti-VEGF groups, even after multivariate analysis. This may have been due to the lack of statistical power from the low number of patients, however, comparison with an untreated cohort (e.g. those who declined treatment) might provide more information.

### Safety concerns: neonatal complications

We identified limited data on neonatal adverse events following intravitreal anti-VEGF administration in pregnant women. Preclinical studies showed that intravitreal bevacizumab injections in rats resulted in adverse developmental effects when administered in early pregnancy, but not in the late stages of pregnancy [15]. None of the live births in our study were noted to have fetal malformations. VEGF plays an important role in regulating physiological processes such as angiogenesis [16], and inhibition of VEGF may confer unknown risks due to its importance in fetal development. It remains unclear whether intravitreal anti-VEGF is safer later in pregnancy.

In contrast, increasing numbers of neonates are being actively treated with anti-VEGF agents for retinopathy of prematurity (ROP). While the results cannot be directly extrapolated to pregnancy, they may provide some limited insights on safety. Follow-up data from the landmark RAINBOW [17] trial of ranibizumab in infants with ROP did not find any correlation between intravitreal ranibizumab injections and neurodevelopmental delay in treated infants up to 2 years later [18, 19]. Caveats include the small sample sizes, lack of longer-term follow-up, and lack of power to fully explore safety outcomes.

### Safety concerns: breastfeeding

Data on breastfeeding in the context of anti-VEGF injections are also very limited [20]. Small pharmacokinetics studies suggest that intravitreal bevacizumab does not result in detectable drug levels in breast milk (*n* = 2) [21]. There are several VEGF isoforms, and one study demonstrated a transient drop in VEGF-A levels in breast milk for the first 24 h after an intravitreal ranibizumab injection, which then recovered to normal levels (*n* = 1) [22]. However, the significance of this is unclear, since conventional infant formula milk does not contain VEGF. For conbercept, an anti-VEGF agent frequently used in China, *n* = 2/3 patients studied did not experience a significant drop in VEGF levels in breast milk [4]. There were no data on serum concentrations of anti-VEGF nor reports of neurodevelopmental evaluations in these children.

### Alternative treatments

CNV carries a risk of permanent severe loss of vision without treatment, and there are no current alternative treatments to anti-VEGF injections for this condition. However, there is no consensus regarding the treatment pathway for DMO in pregnancy [23], and mild to moderate cases of DMO can often be safely observed without treatment. Macular laser can be considered for off-centre DMO, particularly if exudates are tracking towards the centre of the macula. This was not applicable to the patients in our case series. Sometimes DMO improves significantly after pregnancy, without ophthalmic intervention, but for cases where it is severe and vision-threatening, and the risks of observation-only leading to loss of vision outweigh the risks of having ophthalmic treatment, it is worth considering intravitreal treatment options. Intravitreal steroids may be a viable first line therapy but the benefits of this form of treatment must be carefully balanced against the associated risks.

The NICE guidelines previously limited intravitreal dexamethasone implants (Ozurdex) for DMO to pseudophakic patients in the United Kingdom, which would have precluded the majority of women of childbearing age, and hence we were not able to offer Ozurdex to the diabetic patients in our case series. One patient received peri-ocular triamcinolone injection with partial response. With the change to the guidance in 2022, Ozurdex may become an appropriate first-line treatment for pregnant women with DMO – small case series suggest that intravitreal dexamethasone implants may be safe and effective for pregnant women with macular oedema secondary to diabetes [24, 25] or central retinal vein occlusion [26]. However, women need to receive appropriate counselling regarding the risk of needing cataract surgery at a younger age than would typically be the case (up to 60% within 3 years), and the 30% risk of a steroid-induced rise in intraocular pressure, which may be harder to manage given that some intraocular pressure-lowering eyedrops may be relatively contraindicated during pregnancy and breastfeeding [27, 28]. Not all cases of DMO require treatment with anti-VEGF, so there should be a higher threshold for choosing this form of treatment in pregnancy due to uncertainties about risks, and because other treatment options may be available, but a few women may benefit from anti-VEGF injections, and we provide further evidence to enable counselling of these patients.

The patients with DMO in our case series demonstrated good treatment response and subjective improvement in symptoms 4 weeks after receiving anti-VEGF injections, but objective VA gains were limited by structural changes on OCT such as retinal thinning and disorganisation of inner retinal layers that became apparent after the severe DMO had resolved. This highlights the importance of discussing the potentially guarded visual prognosis when counselling such patients on available treatments, but should not preclude them from being offered appropriate treatment.

While consensus guidelines would be useful in guiding the discussion, involving obstetric physicians and/or maternal/fetal medicine obstetricians at all stages of the decision-making process would be of great value in providing high-quality personalised care. Ultimately, it is essential to weigh up the risks of treatment versus long-term vision problems from forgoing treatment, which could affect quality of life (including maintaining vision for driving, reading, working, phone and computer use, and injecting insulin, where relevant) and mental health for these young patients, who should be empowered to make an informed decision.

### Pregnancy testing and counselling

Many patients (40%) in our study were not known to be pregnant at the time of anti-VEGF injection [8, 9, 29–35]. Given the uncertainties around the safety of intravitreal anti-VEGF treatment in pregnancy, we recommend offering pregnancy testing prior to each injection in all women of childbearing potential. This would enable appropriate counselling and informed consent for treatment. A multidisciplinary team approach which includes obstetric physicians, obstetricians, and ophthalmologists is particularly helpful. Deciding on whether to perform a urinary pregnancy test (or relying on patients to report whether they could potentially be pregnant) should be a pragmatic undertaking. For example, sexually active pre-menopausal women of any age should be considered. The purpose of the pregnancy test would be to enable informed consent (as much as possible, given the limited data on anti-VEGF in pregnancy) prior to receiving treatment, rather than serve as a prescriptive rule.

Patients may choose to decline or delay anti-VEGF treatment until after delivery (or breastfeeding) because of concerns about the potential adverse effects of treatment [36, 37]. They should, however, have a detailed discussion of the potential risks, benefits, and alternatives with a knowledgeable clinical team. Strong links between obstetrics, obstetric medicine, and ophthalmology in Oxford have enabled pregnant women with sight-threatening macular pathology to be supported in receiving anti-VEGF treatment. having been appropriately counselled, when they have chosen to do so.

More research is needed on the safety of intravitreal anti-VEGF injections in the peri-conception period, and whether there is a time interval during which pregnancy should be avoided post-treatment. Until further safety data is available, clinicians should consider recommending the use of effective contraception in women of childbearing age in whom anti-VEGF treatments are indicated, also noting that pregnancy tests may be negative very early on in gestation. One patient in the systematic review was inadvertently treated with intravitreal bevacizumab around conception (not known to be pregnant), and experienced a miscarriage 8 weeks later [29]. Given the extended duration of time to miscarriage, and that 10–20% of pregnancies are known to result in miscarriage (with a higher risk in older maternal age, as in this patient), a clear association cannot be drawn [38]. Another patient also received bevacizumab around conception and at 4 weeks’ gestation, but despite multiple risk factors for poor pregnancy outcomes, had a relatively uneventful pregnancy apart from foetal macrosomia in the context of diabetes [33].

### Strengths and limitations

Strengths of this study include a comprehensive case series adding to the body of real-world evidence, combined with a systematic literature review to provide an overview of a relatively uncommon yet challenging issue. The multidisciplinary authorship team including medical retina and obstetric medicine specialists to provide a broad perspective is another strength. There are limitations inherent in any synthesis of case series and reports, including publication bias from unreported cases. There was insufficient longitudinal data on neonatal outcomes such as attainment of neurodevelopmental milestones or ROP screening, which would be of interest in future work. With regards to the case series, limitations include difficulty commenting on the role of pregnancy on DR progression due to previous data being unavailable, such as if patients were referred from external units, and differentiating the impact of pregnancy versus other significant systemic comorbidities in the context of poorly controlled diabetes. However, we hope that this study provides a useful resource for clinicians considering intravitreal anti-VEGF treatment as a means of preserving vision in pregnant women.

## Conclusion

Associations between anti-VEGF and obstetric complications cannot be clearly drawn due to low numbers of patients, as well as multiple confounders which remain to be addressed in future work. Despite this, it was reassuring that in this series of patients, most pregnancies in women knowingly or unknowingly treated with anti-VEGF resulted in uncomplicated pregnancies. Obstetric complications occurred only in the presence of known, significant risk factors for adverse pregnancy outcomes, and the possible miscarriage in the only patient in this series without risk factors occurred exceptionally early in the first trimester. Anti-VEGF treatment did not appear to confer additional risks to neonatal health beyond those related to pre-existing maternal comorbidities, within the constraints of poorly reported data.

We hope these findings will be useful for clinicians treating pregnant women with macular pathology that often requires anti-VEGF treatment, such as CNV. For pregnant women with DMO, where well-established treatments such as macular laser or intravitreal steroid injections may also be considered in some cases, this systematic review shows that anti-VEGF injections are an additional potential treatment option, given the lack of evidence of harm associated with their use.

Close liaison between ophthalmology, obstetric medicine, and obstetric teams is required to inform appropriate counselling on a case-by-case basis. Treatment protocols for intravitreal anti-VEGF injections could potentially incorporate careful history taking regarding pregnancy or breast-feeding status in women of childbearing age and/or urine pregnancy testing, to facilitate informed consent with appropriate counselling and multidisciplinary team support.

## Summary

### What was known before:

- Treating pregnant and breastfeeding women with intravitreal anti-VEGF injections is controversial due to the lack of data on systemic effects on mother and child.
- There are no guidelines or treatment protocols on how to counsel and manage these women.

### What this study adds:

- Clear associations between anti-VEGF and obstetric complications cannot be drawn due to low reported numbers and confounders from high rates of first trimester miscarriages in general and inherently high-risk pregnancies. However, there are limited data to suggest that the judicious use of anti-VEGF injections can provide good visual outcomes without necessarily compromising obstetric ones.
- Many women are inadvertently treated with anti-VEGF injections without being aware of their pregnancy. Treatment protocols for anti-VEGF injections could potentially incorporate careful history taking regarding pregnancy or breast-feeding status in women of childbearing age and/or urine pregnancy testing, to facilitate informed consent with appropriate counselling and multidisciplinary team support.
- Close liaison between ophthalmology and obstetric teams can help facilitate appropriate counselling to enable women to make informed decisions about their treatment.

## Data Availability

All data generated or analysed during this study are included in this published article and its supplementary information files.
